# Identification of Acute HIV-1 Infection by Hologic Aptima HIV-1 RNA Qualitative Assay

**DOI:** 10.1128/JCM.00431-17

**Published:** 2017-06-23

**Authors:** Mark M. Manak, Leigh Anne Eller, Jennifer Malia, Linda L. Jagodzinski, Rapee Trichavaroj, Joseph Oundo, Cornelia Lueer, Fatim Cham, Mark de Souza, Nelson L. Michael, Merlin L. Robb, Sheila A. Peel

**Affiliations:** a U.S. Military HIV Research Program, Walter Reed Army Institute of Research, Silver Spring, Maryland, USA; b Henry M. Jackson Foundation for the Advancement of Military Medicine, Bethesda, Maryland, USA; c Armed Forces Research Institute of Medical Sciences, Bangkok, Thailand; d Walter Reed Project, Kericho, Kenya; e Mbeya Medical Research Centre, Mbeya, Tanzania; f Makerere University Walter Reed Project, Kampala, Uganda; Rhode Island Hospital

**Keywords:** acute HIV-1 infection, Hologic Aptima assay, early HIV-1 infection, HIV-1 RNA

## Abstract

The Hologic Aptima HIV-1 Qualitative RNA assay was used in a rigorous screening approach designed to identify individuals at the earliest stage of HIV-1 infection for enrollment into subsequent studies of cellular and viral events in early infection (RV 217/Early Capture HIV Cohort [ECHO] study). Volunteers at high risk for HIV-1 infection were recruited from study sites in Thailand, Tanzania, Uganda, and Kenya with high HIV-1 prevalence rates among the populations examined. Small-volume blood samples were collected by finger stick at twice-weekly intervals and tested with the Aptima assay. Participants with reactive Aptima test results were contacted immediately for entry into a more comprehensive follow-up schedule with frequent blood draws. Evaluation of the Aptima test prior to use in this study showed a detection sensitivity of 5.5 copies/ml (50%), with all major HIV-1 subtypes detected. A total of 54,306 specimens from 1,112 volunteers were examined during the initial study period (August 2009 to November 2010); 27 individuals were identified as converting from uninfected to infected status. A sporadic reactive Aptima signal was observed in HIV-1-infected individuals under antiretroviral therapy. Occasional false-reactive Aptima results in uninfected individuals, or nonreactive results in HIV-1-infected individuals not on therapy, were observed and used to calculate assay sensitivity and specificity. The sensitivity and specificity of the Aptima assay were 99.03% and 99.23%, respectively; positive and negative predictive values were 92.01% and 99.91%, respectively. Conversion from HIV-1-uninfected to -infected status was rapid, with no evidence of a prolonged period of intermittent low-level viremia.

## INTRODUCTION

An understanding of host-virus interactions in early acute HIV-1 infection (AHI) is critical for the design of effective intervention strategies to reduce rates of HIV-1 transmission and improve long-term outcomes (1–4). Implementation of intervention strategies early in infection, at the time of highest viral burden in blood, semen, and other fluids, can significantly reduce epidemic spread of the infection (5–9). Moreover, initiation of antiviral treatment early in the course of infection may be more effective in disrupting the establishment of stable viral reservoirs and may slow the progression of infection, with possible implications for eventual viral cure (10–12).

A major challenge in the study of early HIV-1 infection is the difficulty in identifying incident cases in sufficient numbers for systematic examination of AHI, typically defined as the time from virus entry to completion of seroconversion (13, 14). Although individuals with AHI frequently develop fever, rash, fatigue, or headache within 2 to 6 weeks after initial exposure, such flu-like symptoms are nonspecific and are often misdiagnosed at initial presentation or simply missed (15, 16). The evolution of HIV-1 screening assays from the first to third generation has allowed for increased sensitivity and therefore earlier detection of HIV-1 antibody in blood (17, 18). The recent introduction of fourth-generation assays, which detect p24 antigen in addition to antibody, has further narrowed the window (period between time of infection and first detection) to less than 2 weeks from infection. The earliest and most sensitive marker of infection, however, is the detection of HIV-1 RNA, which has been successfully used to narrow the window for blood screening to within a few days of infection (19, 20). An approach for even earlier detection of AHI has been the use of pooled nucleic acid testing (NAT) of HIV antibody-negative individuals (21, 22).

The Early Capture HIV Cohort (ECHO) study, RV 217, was implemented in collaboration with researchers in East Africa (Tanzania, Uganda, and Kenya) and Southeast Asia (Thailand) to monitor individuals at high risk of HIV-1 infection, identify acutely infected participants, and collect specimens for the study of early events and progression of infection. In contrast to the U.S. studies, which examined mainly subtype B infections (17), this study encompassed a broad range of HIV-1 subtypes representative of those circulating in the respective regions, including subtypes A, AD, and ACD in Uganda, subtypes A, C, AC, AD, ACD, and AD in Tanzania and Kenya, and subtypes B, CRF01_AE, CRF01_AE/B, and CRF01_AE/C in Thailand (23).

The Aptima HIV-1 RNA qualitative test (Hologic, Inc., San Diego, CA), a U.S. FDA-approved assay for molecular diagnosis of acute and primary HIV-1 infection, was selected for use in this study because of its claims of extreme sensitivity (14 copies/ml) and ability to detect the very earliest times of infection, with reported detection 12 days prior to that of enzyme immunoassay (EIA) repeat-reactive (RR) tests or 6 days prior to that of p24 antigen detection (24, 25). This study evaluated the performance characteristics of the Aptima HIV-1 RNA assay and its ability to detect AHI by twice-weekly testing of small blood volumes (SBVs) from uninfected participants at high risk for HIV-1 infection.

## RESULTS

**FIG 1:**
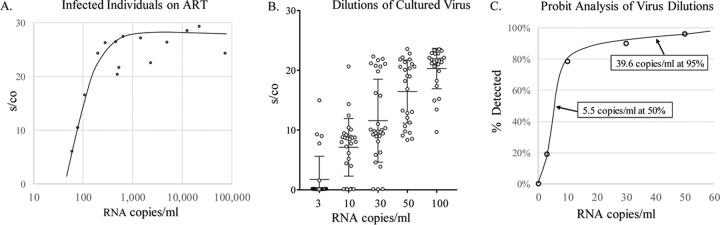
Sensitivity of the Aptima assay. (A) Aptima s/co levels of HIV-1-infected individuals on ART with detectable viral loads plotted against RNA copies/ml as determined by the Roche COBAS AmpliPrep/COBAS TaqMan HIV-1 test. (B) Dilutions of cultured HIV-1 ranging from 3 to 100 copies per ml were tested in 30 replicate measurements. The means and SDs of the resulting s/co ratios on the Aptima assay are shown. (C) Probit analysis of percentage of 30 replicate measurements as described for panel B that yielded a reactive Aptima result at each dilution.

The performance of the Aptima assay was initially evaluated on well-characterized preexisting samples for analytical sensitivity and subtype specificity prior to use in the monitoring of volunteers in RV 217. All HIV-1-negative control samples (*n* = 50) were nonreactive in the Aptima test, with an average signal-to-cutoff (s/co) ratio of 0.12, where values of less than 1.0 are considered nonreactive for a specificity of 100%. All samples (*n* = 33) from HIV-1 antibody-reactive individuals were reactive in the Aptima assay. In addition, all HIV-1-infected individuals (*n* = 20) on antiretroviral therapy (ART) with a detectable viral load (>30 copies/ml) yielded reactive Aptima results, with s/co ratios ranging from 6.5 for samples with very low viral loads to greater than 20 for samples having greater than 500 HIV-1 RNA copies/ml (Fig. 1A).

Limit of detection (LOD) and sensitivity studies were conducted on serial dilutions of precharacterized cultured virus spiked into normal human plasma. Dilutions representing 3, 10, 30, 50, and 100 copies/ml were tested in 30 replicate measurements of the mean plus or minus the standard deviation (SD) of the s/co ratio (Fig. 1B). These results were also used to determine the LOD of the assay based on probit analysis of percent reactive Aptima signal at each dilution (Fig. 1C). Probit analysis indicated 50% detection sensitivity of 5.5 HIV-1 RNA copies/ml and 95% detection sensitivity at 39.6 copies/ml.

**FIG 2:**
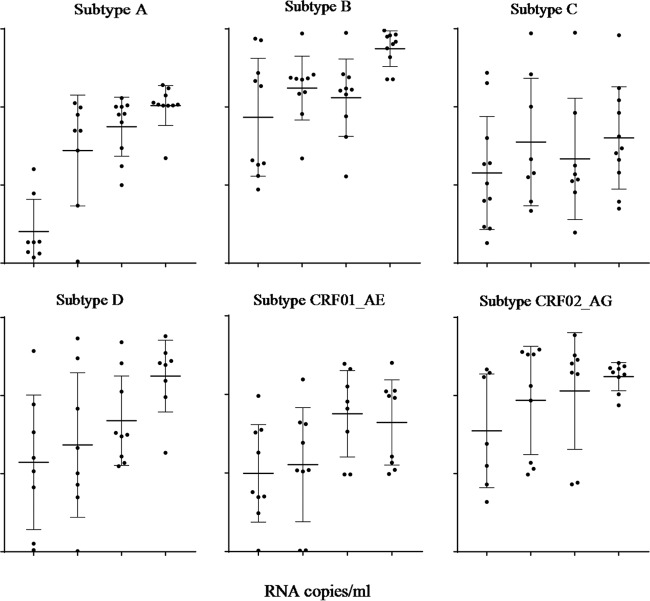
Subtype specificity of the Aptima assay. Dilutions of EDTA-treated plasma spiked with known quantities of HIV-1 virus for subtypes A, B, C, D, CRF01_AE, and CRF02_AG were tested by the Aptima assay. Each dot represents the s/co ratio of a single sample, with each bar showing the mean and SDs.

The HIV-1 subtype specificity and sensitivity of the Aptima assay were also evaluated at various dilutions of cultured viruses representing the major HIV-1 subtypes (Fig. 2). Five replicates of each serial dilution (ranging from 17 to 100 copies/ml) (Abbott m2000 RealTime HIV-1 test) of well-characterized culture supernatants of known HIV-1 subtypes were tested in the Aptima assay. Aptima was capable of detecting all HIV-1 subtypes, with 88%, 90%, and 89% of HIV-1 subtypes A, D, and CRF01_AE, respectively, and 100% of subtypes B, C, and CRF02_AG detected at 17 copies per ml.

**FIG 3:**
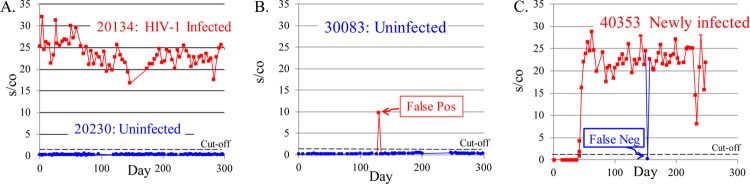
Representative patterns of Aptima reactivity for uninfected and HIV-1-infected participants. (A) HIV-1-uninfected individuals (such as volunteer 20230) show consistently nonreactive Aptima results at each time point over the testing period, while HIV-1-infected individuals (such as volunteer 20134) give consistently high Aptima s/co results. (B) Occasional samples in a presumptive uninfected individual (such as volunteer 30083) yielded a reactive Aptima result, which was nonreactive in repeat or subsequent testing and presumed to be a false-positive result. (C) Occasional samples from an infected individual (such as volunteer 40353) yielded a nonreactive Aptima result that was not confirmed by subsequent tests and was presumed to be false negative. Arrows indicate positions of false-positive and false-negative results.

**FIG 4:**
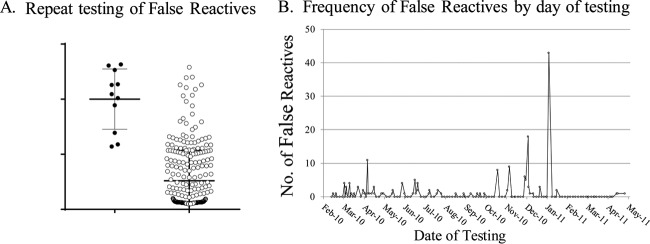
Result of repeat testing of Aptima presumed false-reactive initial results of uninfected participants. (A) Aptima s/co ratios from false-reactive samples in which the repeat sample yielded a reactive or nonreactive result. (B) Clustering of Aptima false-reactive results over time for one site. Average number of assays per day performed during this period was 80 to 100.

**FIG 5:**
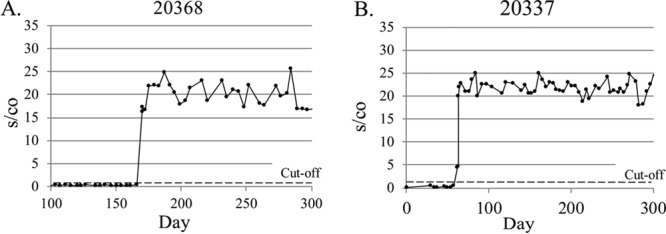
Patterns of Aptima reactivity in acute HIV-1 infection as identified by conversion from nonreactive to reactive for detection of HIV-1 RNA within the period studied. This study permitted identification of very early infection within 3 to 4 days of the first detectable viral RNA.

**FIG 6:**
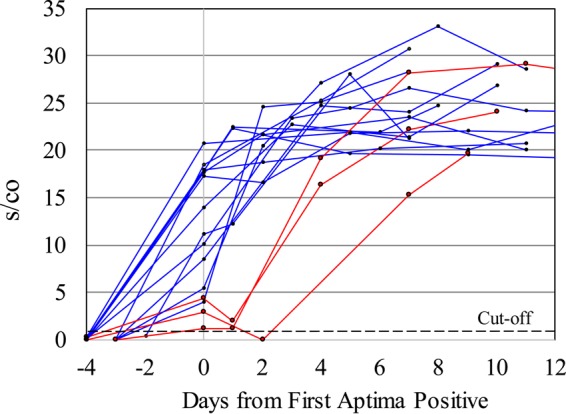
Characterization of the earliest HIV-1 RNA-reactive samples from newly infected individuals. Aptima s/co values immediately prior to and following first Aptima reactivity (defined as day 0). Three individuals had a low or nonreactive Aptima test at days 1 and 2 (in red) before increasing to >15 at the subsequent blood sampling.

A total of 1,112 volunteers, consisting mainly of uninfected individuals at high risk for HIV infection, plus a small subset of HIV-1-infected subjects as controls were enrolled into the surveillance phase of the study and tested twice weekly with the Aptima HIV-1 RNA assay. Typical patterns of Aptima reactivity seen within HIV-1-uninfected and -infected individuals over time are illustrated in Fig. 3 (see also Fig. 5 and 6). Uninfected volunteers (such as volunteer 20230) demonstrated baseline nonreactivity throughout the test period, while infected volunteers (such as volunteer 20134) demonstrated consistently high Aptima s/co ratios that persisted with subsequent SBV testing (Fig. 3A). Occasional samples from uninfected individuals (such as those from volunteer 30083) yielded a reactive signal which was not repeated in testing of subsequent samples and were presumed to represent a false-positive result (Fig. 3B). Conversely, occasional samples from consistently reactive individuals (such as volunteer 40353) for whom an isolated specimen tested nonreactive were presumed to represent a false-negative result (Fig. 3C). When such discrepant results were seen, the same sample was retested the following day whenever possible. In most cases (96.1%) (Fig. 4A), repeat testing of reactive samples from uninfected individuals showed the samples were nonreactive, while repeat testing of all 6/6 repeats of nonreactive samples from infected patients found the samples to be reactive.

Further investigation of false-positive Aptima results is shown in Fig. 4. Of the 383 samples with false-positive results, 285 had sufficient volume for repeat testing. Only 11 of these samples (3.9%) yielded a reactive signal upon retesting of the same specimen, while the remaining samples (96.1%) were negative on retesting. The mean s/co ratio of samples with false-positive results for which repeat testing of the same sample was nonreactive was 4.8, with many samples clustering near cutoff (Fig. 4A), while the mean s/co ratio for the 11 RR samples was 18.6 (*P* < 0.0001). False-positive samples also tended to cluster on certain test dates, as shown for one site in Fig. 4B.

When a first-time-reactive Aptima result was detected, care was taken to confirm that it was, in fact, truly reactive. The sample was retested, if possible, and the subject notified of the reactive result at his or her next visit. Typical patterns of incident infections are shown in Fig. 5. In most cases, such as that of volunteer 20368, a sharp increase in Aptima reactivity was observed, with the s/co ratio increasing to 15 to 20 from the first reactive SBV onward, indicating a clear demarcation between HIV-1-infected and -uninfected status. In some case, the increase occurred over two visits, as was shown for volunteer 20337, whose first reactive Aptima result yielded an s/co ratio just under 5, which increased to greater than 20 by the next visit.

The s/co values of individuals immediately prior to and after the first Aptima detection are shown in Fig. 6. In most cases, a rapid ramp up of RNA reactivity is observed following infection (marked in blue). In three (marked in red) of 27 new infections observed, however, the s/co ratio of the first reactive sample was <5 and subsequent blood samples at day 1 or 2 remained low, and even negative in one case, before increasing to >15 by the next visit, 3 to 5 days later.

**FIG 7:**
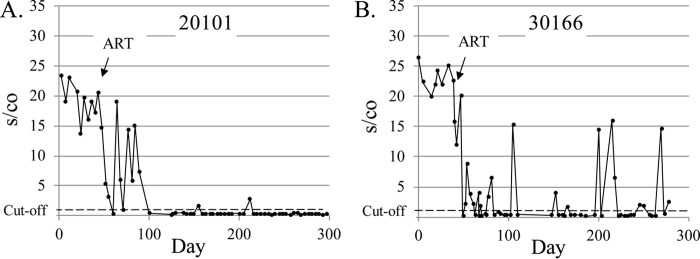
Aptima s/co ratios observed in response to antiretroviral therapy (ART). Volunteer 20101 (A) showed a rapid sustained response to ART with intermittent detectable viremia before achieving virologic control, while volunteer 30166 (B) showed more typical continued sporadic low-level reactivity despite ART therapy. The dotted line represents the Aptima assay cutoff, which is an s/co of greater than or equal to 1.0.

Two examples of Aptima reactivity following initiation of ART are shown in Fig. 7. Participant 20101 showed a rapid decrease in RNA detection to baseline levels within days of initiation of therapy, with a period of sporadic RNA reactivity persisting in the first few weeks posttherapy, and near cutoff, but reactive signal observed occasionally at subsequent periods. Sporadic “spikes” of low-level RNA signal, such as seen for volunteer 30166, were frequently observed in all 15 volunteers under ART therapy examined during this period.

The sensitivity and specificity of the Aptima assay were also evaluated based on the false-positive and false-negative rates observed in screening these high-risk populations. For the purposes of this analysis, we defined a false-positive signal as a reactive result in an otherwise HIV-1-uninfected volunteer and a false-negative as a nonreactive result in a persistently Aptima-reactive individual (as illustrated in Fig. 3B and C). Aptima results at the time of transition from nonreactive to reactive in newly infected individuals and in individuals on ART were excluded from these calculations. Of 54,306 observations from volunteers whose results were consistently nonreactive over time, a total of 383 of 4,795 positive results were potentially false positive (7.99%), as they were not confirmed by testing of subsequent blood samples (Table 1). In contrast, the false-negative results (43/49,511; 0.09%) observed during this period were much lower. The data also allowed evaluation of the sensitivity and specificity of the assay based on consensus Aptima test results on subsequent samples from the same individual as the gold standard. Using these criteria, the sensitivity and specificity of the Aptima assay were 99.03% and 99.23%, respectively. The corresponding positive predictive value (PPV) and negative predictive value (NPV) were 92.01% and 99.91%, respectively, for testing of high-risk populations.

**TABLE 1:** False-positive and false-negative Aptima results^*a*^

Aptima test result (*n*)^*b*^	Consensus test result (*n*)		
	Positive	Negative	Total
Positive	4,412	383	4,795
Negative	43	49,468	49,511
Total	4,455	49,851	54,306

a The sensitivity and specificity (95% confidence interval) were 99.03% (98.70–99.30%) and 99.23% (99.15–99.31%), respectively, and the PPV and NPV (95% confidence interval) were 92.01% (91.25–92.72%) and 99.91% (99.88–99.94%), respectively.

b The numbers of false-positive results (uninfected individuals scoring reactive) and false-negative results (HIV-1-infected individuals scoring nonreactive) are shown; the consensus of subsequent testing was used as the gold standard.

## DISCUSSION

Previous studies, such as those based on sequential screening of plasma donors at blood banks (17), demonstrate that HIV-1 RNA is the earliest detectable marker of infection. The approach presented for systematic identification of specimens at the earliest stage of HIV-1 infection was based on frequent monitoring of high-risk populations by the very sensitive Aptima HIV-1 RNA test on SBV samples that can be quickly and easily collected. The use of minimally invasive finger-stick sampling allowed volunteers to come in on a twice-weekly basis for a brief visit that minimized disruption of routine daily schedules. The low LOD of the Aptima assay (5.5 RNA copies/ml at 50% level) permitted testing of a 1:5 dilution of sample yet remained within the detectable range based on previously reported viral load results for early samples (17). The frequent testing ensured infection detection within days of initial infection, well before the appearance of anti-HIV antibody responses. The wide subtype specificity ensured reliable results in all the geographical areas assessed. The excellent sensitivity (99.03%) and specificity (99.23%) of this assay were reaffirmed on sequential serial samples as a gold standard for infection classification assessment during the course of this study. Patterns of HIV-1 RNA nonreactivity and reactivity indicative of HIV-1-uninfected, chronically infected, infected individuals on ART, and newly infected individuals are presented in this paper.

Previous studies focused on acute HIV-1 infection experienced limited success in detecting sufficient numbers of cases for a systematic evaluation as described in this paper. A previous attempt to identify AHI in a high-risk population of men who have sex with men (MSM) by specifically targeting individuals with fever or flu-like symptoms who presented for HIV testing failed to identify a higher rate of acute infections (26). Rather than relying on symptoms, greater success was achieved using pooled nucleic acid testing (NAT), which demonstrated enhanced performance in detecting acute infections (27–32). Screening of 13,226 individuals by a finger-stick rapid antibody test followed by pooled NAT testing of HIV-1-seronegative individuals identified 115 HIV antibody-positive individuals and an additional 8 cases identified by pooled NAT, for an AHI detection rate of 0.12% (30). A similar pooled NAT study of 65,220 individuals at sexually transmitted infection clinics in New York City also showed a 0.06% detection of AHI, with 40 AHI cases detected (22). Additional studies in Dallas and North Carolina and at multisites identified acute HIV cases in 0.06% to 0.17% of individuals screened (28, 29, 31). The low incidence of HIV in the populations examined and the very brief time interval of acute infection led to very large numbers of samples screened to yield relatively low rates of acute infection cases. Use of pooled NAT in a high-risk Thai population improved detection of AHI by 38% relative to fourth-generation assays: 81 detected by fourth-generation assays and 112 by pooled NAT (37). In contrast to the U.S. studies, our studies in Africa and Thailand have successfully identified AHI in 2.0 to 5.1% of the individuals screened, with successful identification and recruitment of high numbers of AHI cases (23). The success of this study in detecting AHI is a combination of targeting a high-risk population, frequent sampling using a minimally invasive finger-stick approach, and testing with a highly sensitive assay for HIV-1 RNA.

The limited plasma volume from finger-stick samples collected during the surveillance phase required selection of an assay with high sensitivity. The Aptima assay met this requirement, as a 5-fold dilution of low HIV-1 RNA-concentration specimens (5.5 copy/ml) in our laboratory would correspond to a detection sensitivity of ∼27.5 copies/ml (Fig. 1). The Aptima product insert reports a 98.5% detection sensitivity of 30 HIV-1 RNA copies/ml (32); thus, dilution of low concentration specimens would not be expected to compromise detection. Quantitative HIV-1 RNA assays, such as the Roche COBAS AmpliPrep/COBAS TaqMan HIV-1, v2.0, and Abbott RealTime HIV-1 were also considered for use in participant screening. The higher input volume requirements for these assays necessitate a 12-fold dilution. With reported package insert LODs of 20 and 40 HIV-1 RNA copies/ml (33), the corresponding LODs would be ∼240 and ∼480 copies/ml for the Roche and Abbott assays, respectively; thus, these assays were not found suitable for this unique study. The number of assays performed permitted evaluation of the sensitivity and specificity of the Aptima test in the context of high-risk populations. Our definition of a gold standard for infection based on results of closely spaced, sequential, serial blood samples from the same individual have shown that false-negative results were relatively rare (0.09%), while false-positive results could occur at relatively high frequency (7.99%) in these high-risk populations. The false-positive results tended to have low s/co ratios; 97% were nonreactive with repeat testing. About half of all false-positive results tended to cluster on specific dates at specific sites, suggesting an assay performance issue such as inadequate washing, carry-over contamination, and/or technician error. The very small number (11) of false-positive results for which a repeat reactive result was obtained are therefore more likely due to laboratory error, although we cannot rule out that these samples represent very short transient exposure to the virus that failed to establish infection. The relatively high rates of false-positive Aptima results when used in the context of this study for screening high-risk populations emphasize the need for confirmatory testing to ensure correct infection status classification.

In the majority of volunteers, the transition from Aptima nonreactive to reactive in newly infected individuals was rapid, with a consistent rate of increase in s/co values in early acute infection. Only three of 27 (3/27) incident infections showed low-level viremia for two consecutive dates in earliest infection. Thus, this study did not find intermittent low-level HIV-1 viremia preceding early AHI as previously reported (34), but is more consistent with a subsequent report of no reproducible transient viremia prior to infection (35). The sporadic HIV-1 RNA reactivity observed in volunteers on ART may represent residual low-level viremia detected by the very sensitive Aptima assay, which has the potential for increased sensitivity compared to the quantitative assays. Intermittent low-level viremia (blips) in patients on retroviral therapy may represent inadequate adherence to the prescribed drug regimens or early indicators of virologic failure (36).

This study demonstrated that frequent sampling of high-risk populations by testing of small volume finger-stick samples by Aptima was a successful approach to identification of individuals at the earliest stages of HIV-1 infection. Identification of AHI has allowed us to collect specimens for the study of early events in HIV-1 infection and provides an invaluable resource for future study. Early detection of HIV-1 infection may also permit studies of earlier application of intervention therapies to block establishment of stable viral reservoirs and evolution of infection (10, 37–39).

## MATERIALS AND METHODS

### Characterization of Aptima assay sensitivity and specificity.

Initial evaluation of assay sensitivity and specificity was performed on residual clinical specimens from previously tested individuals, including 50 plasma samples from healthy, uninfected individuals; 33 HIV-1 first time antibody-reactive specimens from HIV screening, and 20 clinical specimens from HIV-1-infected patients under therapy with known viral load levels measured by the Roche COBAS AmpliPrep/COBAS TaqMan HIV-1 Test v1.0.

### Limit of detection (LOD).

Serial dilutions of a stock subtype B isolate (84US_MN) in EDTA-treated plasma were generated to yield final titers of approximately 100, 50, 30, 10, and 3 copies/ml and tested in six replicates each over 5 runs (total of 30 replicates). LOD, defined as the viral load detected at 95% and 50% confidence intervals, was determined by probit analysis.

### Subtype specificity and sensitivity.

Serial dilutions of each of six major HIV-1 subtypes, A, B, C, D, CRF01_AE, and CRF02_AG, (40) were prepared targeting concentrations of 100, 50, 25, and 17 copies/ml based on quantitation of the respective stocks by the Abbott m2000 RealTime assay and tested in duplicate in the Aptima assay.

### Study population and testing.

The present study represents the analysis of 1,112 high-risk individuals enrolled between October 2009 and November 2010 in Kenya, Tanzania, Uganda, and Thailand and includes men and women, aged 18 to 50 years old, at high risk for HIV-1 infection with an overall prevalence rate of 28.4% in the study population (23). A small number of prevalent HIV-1-infected individuals were also enrolled for masking to minimize the risk of stigmatization. HIV serology was performed at screening and subsequently at 6-month intervals using the Bio-Rad Genetic Systems HIV-1/HIV-2 Plus O EIA (Bio-Rad Laboratories Inc., Redmond, WA). Reactive samples were repeated in duplicate then confirmed by the Genetic Systems HIV-1 Western blot (Bio-Rad Laboratories). SBV samples were obtained via finger stick twice weekly for testing in the Aptima HIV-1 RNA assay. Following a reactive test, volunteers entered the second part of the study where larger volumes of blood were collected every 3 to 4 days for 4 weeks, and HIV-1 infection was confirmed via routine HIV-1 testing described above. Individuals with confirmed HIV-1 infection were then followed for 5 years.

### HIV-1 Aptima testing.

During the surveillance phase, approximately 600 μl of whole blood from finger sticks was collected into a BD Microtainer (Becton Dickinson, San Jose, CA) containing EDTA. Whole blood was centrifuged at 9,000 × *g* for 3 min. Plasma was diluted 1:5 in phosphate-buffered saline and tested for HIV-1 RNA using the Aptima HIV-1 RNA qualitative assay. Quantitative plasma HIV-1 RNA determinations were performed by the RealTime HIV-1 assay (Abbott Laboratories, Abbott Park, IL) on the m2000 platform.

### Statistical analysis.

Means and standard deviations were obtained from and *t* test and other statistical analyses were performed on the GraphPad v7.01 software from Prism. Probit analyses at 50% and 95% confidence intervals were run on SAS software. Sensitivity, specificity, and positive predictive values were calculated using MedCalc software.

### Ethical considerations.

All study participants were enrolled after completing an Institutional Review Board (IRB)-approved informed consent process. HIV counseling and testing were offered throughout the study. The study was approved by the Walter Reed Army Institute of Research (WRAIR) IRB and by the National Health Research Ethics Committee of the respective countries. Specimens were transported to the United States for further testing as specified in the IRB-approved study protocol under approved materials transfer agreements (MTAs).
